# Resistance Exercise Regulates Hepatic Lipolytic Factors as Effective as Aerobic Exercise in Obese Mice

**DOI:** 10.3390/ijerph17228307

**Published:** 2020-11-10

**Authors:** Ju Yong Bae

**Affiliations:** Department of Physical Education, Dong-A University, Busan 49315, Korea; kosa99@dau.ac.kr

**Keywords:** treadmill exercise, ladder climbing, regular exercise, training, hepatic steatosis

## Abstract

Non-alcoholic fatty liver disease (NAFLD) is associated with obesity. The effect of resistance exercise without dietary restriction on the regulation of hepatic lipolytic factors is unclear. This study aimed to analyze the effects of aerobic and resistance exercise on hepatic lipolytic factors of obese mice. High-fat diet (HFD)-induced obese mice were divided into HFD + sedentary (HF), HFD + aerobic exercise, and HFD + resistance exercise groups. Exercise group mice were subjected to treadmill or ladder climbing exercise for 8 weeks. Fat mass and liver triglycerides were significantly decreased in both aerobic and resistance training groups. In the results of protein levels related to hepatic steatosis, HFD significantly increased liver cannabinoid receptor 1 and sterol-regulatory element binding protein 1 (SREBP-1). Both aerobic and resistance training significantly (*p* < 0.05) increased liver carnitine palmitoyltransferase-1, phosphor-AMP-activated protein kinase (p-AMPK), and p-AMPK/AMPK and decreased liver SREBP-1. However, the type of exercise did not exert any significant effects on these protein levels. Thus, resistance exercise, similarly to aerobic exercise, effectively regulated hepatic lipolytic factors of obese mice. Therefore, a sustainable type of exercise selected based on the fitness level, disease type, musculoskeletal disorder status, and preference of the patients is the best exercise intervention for alleviating NAFLD.

## 1. Introduction

Consumption of a chronic high-fat diet (HFD) increases body weight and fat mass, which is a well-known powerful factor of obesity. Excess nutrients from dietary intake are stored in the form of lipid droplets in adipose tissue, but if the storage capacity of adipose tissue is exceeded, increased fat accumulation occurs in other tissues, including the liver, muscle, and heart [1,2]. Excessive lipid accumulation in non-adipose tissues can lead to cellular dysfunction, lipotoxicity, and cell death [3].

Non-alcoholic fatty liver disease (NAFLD) is closely associated with obesity [4] and has currently emerged as the most common chronic liver disease [5]. NAFLD prevalence in Asian populations is around 25%, which is similar to the rate in many western countries [6]. Even though hepatocellular carcinoma and end-stage liver disease, which are secondary to NAFLD, rarely occur, NAFLD has shown a rising trend in prevalence [7] and an increase in mortality [8].

Body weight reduction by at least 3–5% is essential to alleviate NAFLD, since NAFLD occurrence is closely related to obesity and increased body fat mass [9,10]. Therefore, lifestyle modifications, such as exercise and dietary restriction, which significantly reduce body weight, have been recommended as the most effective intervention for NAFLD [11]. Particularly, aerobic exercise is a commonly recommended treatment for NAFLD because it exerts beneficial effects, including weight loss, by increasing energy consumption [9,12].

However, the results of several animal and human studies have suggested that regular exercise may also be effective in alleviating NAFLD, even without weight loss. Some previous studies reported that exercise without dietary or caloric restriction did not cause weight loss [13,14]. Although there was no benefit of weight reduction, regular aerobic exercise increased the lipolytic protein levels in the liver and improved glucose tolerance in HFD-induced obese mice [13]. A previous human study also reported that aerobic exercise for 4 weeks led to a decrease in hepatic triglyceride (TG) concentration and visceral adiposity in obese individuals without weight loss [14]. In this respect, resistance exercise through repetitive muscle contraction and relaxation may have a positive effect on an increase in hepatic lipolysis in obese individuals, although resistance exercise is not an effective strategy for weight loss. In addition, most studies on alleviating NAFLD have focused on the effect of aerobic exercise and/or dietary restriction, but the effects of resistance exercise, a form of muscular endurance exercise, on hepatic lipolysis remain unclear.

Therefore, this study aimed to analyze the effects of aerobic and resistance exercise on hepatic lipolytic factors in HFD-induced obese mice without dietary restriction.

## 2. Materials and Methods

### 2.1. Animals

Forty male C57BL/6 (4-week-old) mice were purchased from Samtako Bio Korea (Osan, Korea) and fed freely with standard chow and water until they turned 30 weeks old. Three or four mice were housed in each cage and maintained under standardized conditions in an animal facility of Dong-A University College of Medicine. Relative humidity, temperature, and a 12 h dark–light cycle were constantly maintained. This study was approved by the Dong-A University Medical School Institutional Animal Care and Use Committee (DIACUC-approval-19-11).

### 2.2. Experimental Design

When the mice turned 30 weeks old, they were randomly divided into two groups: normal diet (CO, *n* = 10) and HFD (*n* = 30) groups. The CO group mice were fed with a standard chow (69.4% carbohydrate, 6.3% lipid, and 24.3% protein), whereas the HF group mice were fed with a 60% fat chow (20% carbohydrate, 60% lipid, and 20% protein) to induce obesity for 8 weeks. Thereafter, the HF group mice were subdivided into HFD + sedentary (HF, *n* = 10), HFD + aerobic training (HFAT, *n* = 10), and HFD + resistance training (HFRT, *n* = 10) groups, and the training groups were subjected to exercise for 8 weeks. Body weight was measured every week until the end of the experimental period.

### 2.3. Training Intervention

The aerobic and resistance training group mice were subjected to moderate-intense exercise, which is well known as an effective exercise intensity for alleviating the NAFLD, 5 days per week for 8 weeks, while maintaining the HFD. During week 1, mice were subjected to low-intensity exercise to allow them to adapt to the exercise equipment and set the exercise intensity. Thereafter, mice were subjected to exercise at a determined speed or resistance for the remaining 7 weeks. The CO and HF group mice were placed near the treadmill to expose them to a stress condition similar to that exposed to the training group mice when they were subjected to exercise.

Specifically, the HFAT group mice were subjected to exercise 5 days per week for 8 weeks on an animal treadmill [15]. During the first four days of week 1, mice were subjected to aerobic exercise at a speed of 5 m/min for 5 min on day 1, and the exercise time was increased by 5 min every day until day 4. On day 5 of week 1, an incremental load test was performed to determine the treadmill exercise speed for the HFAT group. Briefly, after a 5-min warm-up at 5 m/min, the treadmill speed was increased by 3 m/min every 3 min at 0% grade until exhaustion. The exercise speed for the HFAT group was set to 65~70% of the maximal speed based on the incremental load test results. Mice were subjected to exercise 50 min per day, including 10 min of warm-up and 10 min of cool-down exercise, for 5 days per week for the remaining 7 weeks.

The resistance exercise program referred to the ladder climbing exercise protocol of the previous study [16]. The HFRT group mice were subjected to exercise 5 days per week for 8 weeks on a climbing ladder (a 100-cm ladder with a 2-cm grid and 80° incline). A house chamber (L × W × H = 25 × 25 × 20 cm) was set up on top of the ladder for a 2-min rest period during the trial. During the first four days of week 1, mice were subjected to exercise without any resistance to allow them to adapt to the ladder. On day 5 of week 1, an incremental load test was conducted, as described in a previous study, to determine the exercise intensity [16]. Briefly, 50% of the body weight was attached to the mouse’s tail (lead), and the lead weight was progressively increased by 3 g when the mouse successfully climbed the ladder. Mice, with 65~70% of the maximum weight based on the incremental load test results, were subjected to eight sets of exercise for the remaining 7 weeks.

### 2.4. Tissue Sampling

Tissue sampling was conducted 2 days after the last exercise session to avoid any temporary training effects. Food was discontinued 12 h before sacrifice. Epididymal white adipose tissues and liver tissues were excised under anesthesia using ethyl ether. The samples were immediately frozen and stored at −80 °C.

### 2.5. Hematoxylin and Eosin (H&E) Staining

Liver tissue was fixed with 10% NBF buffer and embedded in paraffin. Samples were cut into 4 µm slides, which were then stained with H&E. Digital images were captured using an Aperio ScanScope (Leica Biosystems, Richmond, IL, USA).

### 2.6. Extraction of Liver TGs

As previously described [13], liver tissue (50 mg) was collected in an e-tube that contained 200 µL of ethanol KOH (2 parts ethanol: 1 part 30% KOH) and incubated overnight at 55 °C. Subsequently, 50% ethanol was added to obtain a total volume of 0.5 mL, and the resulting mixture was centrifuged at 13,000 rpm for 5 min. The supernatant was transferred into a new tube, and 50% ethanol was added to obtain a total volume of 0.6 mL. After vortex mixing, the mixture (200 μL) was transferred to a new e-tube, and 1 M MgCl_2_ (215 µL) was added. Then, the mixture was incubated on ice for 10 min and centrifuged at 13,000 rpm for 5 min. The supernatant was transferred into a new tube and incubated at 37 °C for 10 min. Liver TG was then analyzed by the enzymatic colorimetric method using Triglyceride Reagent (Asan Pharmaceutical, Seoul, Korea). The absorbance at 550 nm was measured using a microplate spectrophotometer (TECAN sunrise, Tecan GmbH, Salzburg, Austria).

### 2.7. Protein Analysis

As previously described [15], the liver tissue was lysed in RIPA buffer (200 µL). Thereafter, the samples were homogenized and centrifuged at 14,000 rpm for 30 min. The protein concentration was measured by the bicinchoninic acid method. Samples were resolved by SDS-PAGE and transferred to polyvinylidene difluoride membranes. The membranes were blocked with 5% non-fat dry milk in phosphate-buffered saline. Subsequently, the membranes were incubated overnight at 4 °C with primary antibodies (1:1000 dilution) against cannabinoid receptor 1 (CB1, Catalog No. sc-293419; Santa Cruz Biotechnology, Dallas, TX, USA), sterol-regulatory element binding protein 1 (SREBP-1, Catalog No. sc-13551, Santa Cruz Biotechnology), carnitine palmitoyltransferase 1 (CPT1, Catalog No. sc-98834, Santa Cruz Biotechnology), AMP-activated protein kinase (AMPKα, Catalog No. #2532; Cell Signaling Technology, Danvers, MA, USA), and phosphor-AMPK (p-AMPK, Catalog No. #2531; Cell Signaling Technology) and then secondary antibodies (1:5000 dilution) for 1 h. Finally, the fluorescent signal was developed using an enhanced chemiluminescence solution and visualized using the ImageQuantTM LAS-4000 System (GE Healthcare, Uppsala, Sweden).

### 2.8. Statistical Analysis

All statistical analyses were performed with the SPSS v22.0 (SPSS Inc., Chicago, IL, USA), and the values are reported as the mean ± standard error. Two-way repeated measures analysis of variance (ANOVA) was used to compare the changes in body weight, and one-way ANOVA, followed by Tukey HSD (honestly significant difference) post hoc test, was conducted to compare the differences between the groups. The effect size (ES) was calculated for each variable using partial eta squared. The homogeneity of variance of all data was confirmed by Levene’s test, and statistical significance was set at 0.05.

## 3. Results

### 3.1. Changes in Body Weight and Fat Mass after Obesity Induction and Exercise Intervention

**Figure 1:**
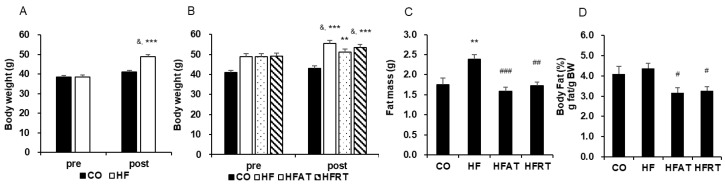
Changes in body weight and body fat after obesity induction and exercise intervention. Changes in body weight after 8 weeks of high-fat diet (HFD) (**A**) and 8 weeks of exercise (**B**) are presented. Changes in fat mass (**C**) and % body fat (**D**) after 8 weeks of exercise are also presented. Data are expressed as the mean ± standard error. CO, normal diet group; HF, HFD group; HFAT, HFD + aerobic training group; HFRT, HFD + resistance training group; BW, body weight; ^&^ *p* < 0.001, versus before; *** *p* < 0.001, versus the CO group; ** *p* < 0.01, versus the CO group; ^#^ versus HF group, *p* < 0.05; ^##^ versus HF group, *p* < 0.01; ^###^ versus HF group, *p* < 0.001.

Obesity was induced in the HF group mice by the HFD, as shown by the significant increase in the body weight of the HF group mice (Figure 1A). A significant difference across groups by time interaction was observed after 8 weeks of obesity induction (F = 67.278, *p* < 0.001, ES = 0.639). Post-hoc analysis showed significantly increased body weight of the HF group mice after 8 weeks of HFD (t = 19.501, *p* < 0.001). Besides, body weight was significantly higher in the HF group than in the CO group after 8 weeks of HFD (t = 6.000, *p* < 0.001).

Although HFD induced an increase in body weight, aerobic training intervention prevented this increase in body weight (Figure 1B). A significant difference across groups by time interaction was observed after 8 weeks of exercise intervention (F = 7.963, *p* < 0.001, ES = 0.399). Although the body weight of the CO and HFAT group mice did not increase, the body weight of the HF (t = 5.374, *p* < 0.001) and HFRT (t = 9.786, *p* < 0.001) group mice was significantly increased after exercise intervention. Besides, body weight was significantly higher in the HF (*p* < 0.001), HFAT (*p* < 0.01), and HFRT (*p* < 0.001) groups than in the CO group after exercise intervention (F = 12.495, *p* < 0.001, ES = 0.510).

Fat mass was significantly higher in the HF group than in the CO group (*p* < 0.01), but that was significantly lower in the HFAT (*p* < 0.001) and HFRT (*p* < 0.01) groups than in the HF group after exercise intervention (F = 8.959, *p* < 0.001, ES = 0.427) (Figure 1C). Besides, % body fat of the HFAT (*p* < 0.05) and HFRT (*p* < 0.05) group mice was significantly lower than that of the HF group (F = 4.195, *p* < 0.05, ES = 0.259) (Figure 1D).

### 3.2. Changes in the Liver Weight, Liver Triglycerides, and Hepatic Lipid Droplet after Exercise Intervention

**Figure 2:**
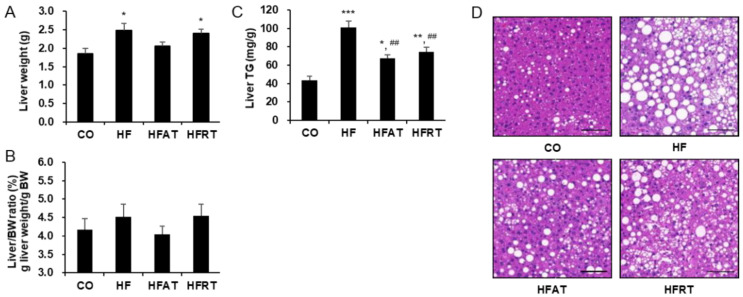
Changes in the liver weight, liver triglycerides (TG), and hepatic lipid droplet after exercise intervention. Changes in the liver weight (**A**), liver/BW ratio (**B**), liver TG levels (**C**), and hepatic lipid droplet (**D**) after exercise intervention are presented. Data are expressed as the mean ± standard error. CO, normal diet group; HF, high-fat diet (HFD) group; HFAT, HFD + aerobic training group; HFRT, HFD + resistance training group; BW, body weight; * *p* < 0.05, versus the CO group; ** *p* < 0.01, versus the CO group; *** *p* < 0.001, versus the CO group; ^##^ *p* < 0.01, versus the HF group. Scale bar = 20 µm.

Liver weight was significantly higher in the HF (*p* < 0.05) and HFRT (*p* < 0.05) groups than in the CO group (F = 5.164, *p* < 0.01, ES = 0.301) (Figure 2A), but there was no statistically significant difference in the liver/body weight (BW) ratio (Figure 2B). Despite no difference in liver/BW ratio, liver TG was significantly higher in the HF (*p* < 0.001), HFAT (*p* < 0.05), and HFRT (*p* < 0.01) groups than in the CO group (F = 21.014, *p* < 0.001, ES = 0.724). However, liver TG in the training groups was significantly lower than that in the HF groups (*p* < 0.01) (Figure 2C). Hepatic lipid droplets in the HF group increased, but that in the exercise group decreased (Figure 2D).

### 3.3. Protein Levels Associated with Hepatic Lipolysis after Exercise Intervention

**Figure 3:**
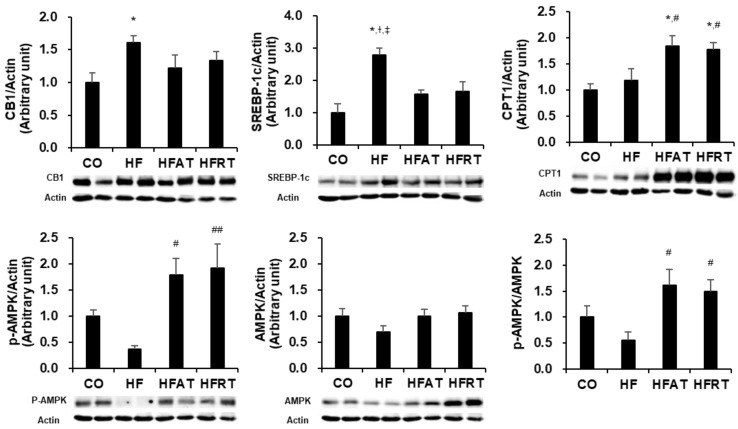
Protein levels associated with hepatic lipolysis after exercise intervention. Data are expressed as the mean ± standard error. CB1, cannabinoid receptor 1; SREBP-1c, sterol-regulatory element binding protein 1; CPT1, carnitine palmitoyltransferase 1; p-AMPK, phosphor-AMP-activated protein kinase; CO, normal diet group; HF, high-fat diet (HFD) group; HFAT, HFD + aerobic training group; HFRT, HFD + resistance training group; * *p* < 0.05, versus the CO group; ^#^ *p* < 0.05, versus the HF group; ^##^ *p* < 0.01, versus the HF group; ^†^ *p* < 0.05, versus the HFAT group; ^‡^ *p* < 0.05, versus the HFRT group.

Protein levels associated with hepatic lipolysis after 8 weeks of exercise intervention are presented in Figure 3. Following 8 weeks of exercise intervention, CB1 was significantly higher in the HF group than in the CO group (*p* < 0.05, ES = 0.260), and SREBP-1c was significantly higher in the HF group than in the other groups (*p* < 0.05, ES = 0.546). p-AMPK (*p* < 0.05, ES = 0.442) and p-AMPK/AMPK (*p* < 0.05, ES = 0.354) were significantly higher in the training groups than in the HF group, and CPT1 was significantly higher in the training groups than in the CO and HF groups (*p* < 0.05, ES = 0.444).

## 4. Discussion

This study aimed to analyze the effects of aerobic and resistance exercise without dietary restriction on hepatic lipolysis of HFD-induced obese mice. The results of this study revealed that 16-week chronic HFD led to a significant increase in body weight and induced hepatic steatosis. However, both regular aerobic and resistance exercises were effective in improving fat mass and hepatic lipolysis even without weight reduction.

Regular exercise, along with physical activity and diet, is well documented as the most effective intervention for the preventive and therapeutic treatment of chronic and adult diseases [17,18]. Exercise exerts different effects depending on not only individual characteristics such as age, gender, and disease, but also exercise characteristics such as frequency, intensity, type, and time [18,19]. However, exercise is an intervention without side effects and cost burden for chronic diseases, and the effects of exercise are mainly positive [20].

Aerobic exercise mainly improves the cardiovascular system by increasing peak oxygen consumption, whereas resistance exercise stimulates the neuromuscular system through repetitive contraction and relaxation of specific muscles [21]. Exercise-induced activation of the cardiovascular and muscular systems increases energy consumption during and after exercise. Most of the energy needed to produce ATP at rest comes from fatty acid oxidation [22], resulting in body weight and fat reduction, which is the most powerful effect of exercise [23]. In this study, both aerobic and resistance exercises inhibited the increase in adipose tissue, even though HFD was maintained. High abdominal visceral fat level, which increases insulin resistance and inflammation through adipokine secretion, is an important risk factor of chronic inflammation and metabolic disorders [24,25]; thus, exercise-induced reduction in abdominal visceral fat mass has been suggested as a measure of health improvement. As expected, aerobic exercise was effective in suppressing the increase in fat mass in HFD-induced obesity, regardless of dietary changes. Although there was no benefit of weight reduction due to no dietary changes to incorporate a low-calorie or low-fat diet, the results of this study are noteworthy because they showed that regular resistance exercise also suppressed fat accumulation.

NAFLD is the early stage of liver disease but is also very dangerous due to the possibility of metastasis to secondary disease and its involvement in the systemic energy metabolism [26]. Sedentary lifestyle and excessive nutrient intake induce obesity, which quickly leads to hepatic steatosis; therefore, weight loss through lifestyle modifications in liver disease patients has been suggested as the priority goal for alleviating hepatic steatosis [9]. For this reason, studies on dietary conversion [27] and combined treatment of exercise and dietary conversion [28], which are effective for weight loss, instead of only exercise treatment, have been actively studied. However, sudden or quick weight loss through dietary changes may lead to a more serious stage of NAFLD [29]. Additionally, losing weight is never an easy task for obese people and patients. Therefore, a systematic approach of lifestyle modifications must be applied to mitigate NAFLD. In this respect, moderate-intensity aerobic and resistance exercise were conducted in HFD-induced obese mice in this study. We found that both aerobic and resistance exercise improved liver TG levels and hepatic lipid droplet. The fundamental reason for the occurrence of hepatic steatosis is an imbalance between fat input and output in the liver [30,31]. Diet regulation contributes more to reducing the fat input in the liver, while exercise contributes to reducing the fat output in the liver by regulating hepatic β-oxidation and lipogenesis [32]. Therefore, the results of this study showed that the increase in fat input due to continuous HFD induction was balanced by the increase in fat output due to regular exercise intervention to a certain extent.

Indeed, the results of this study showed that both aerobic and resistance exercise alleviated hepatic steatosis in obese mice by regulating hepatic fat accumulation-related factors, such as CB1, SREBP-1, CPT1, and AMPK. It is well established that aerobic exercise increases lipolysis in the liver tissue and alleviates NAFLD. Ok et al. [13] reported that 8 weeks of treadmill exercise improved glucose tolerance and hepatic steatosis due to an increase in lipolytic factors, such as p-AMPK and CPT1. Another study reported that 8 weeks of treadmill running alleviated hepatic steatosis by increasing fatty acid oxidation (CPT1 mRNA and p-AMPK/AMPK) and suppressing lipogenic factors (SREBP-1c mRNA) [33]. Additionally, aerobic exercise causes adenosine acetyl-CoA production by upregulating β-oxidation, and this acetyl-CoA generates ATP in the mitochondrial electron transport system [34]. However, the effector mechanism of resistance exercise in reducing hepatic steatosis remains unclear. One possible explanation is that resistance exercise produces specific intramuscular proteins called myokines, such as Meteorin-like (Metrnl) and Irisin, and skeletal muscles interact with other organs through the transport of these myokines [35,36]. Rao et al. [35] suggested that resistance exercise upregulated peroxisome proliferator-activated receptor γ coactivator-1α (PGC-1α), resulting in an increase in Metrnl in muscle tissues. The upregulated Metrnl in skeletal muscle then transferred the positive effects of PGC-1α onto the other tissues [35]. Boström et al. [36] also reported that skeletal muscles interact with other organs by secreting a myokine called “Irisin”. Overexpression of intramuscular Irisin reduces hepatic steatosis by inhibiting lipogenic factors, such as SREBP-1 and FAS, in hepatocytes [37]. Therefore, the interaction of muscles with other organs might partially explain the effects of resistance training on hepatic steatosis reduction. In this study, muscle-specific proteins and organs (other than muscles) that might interact with the liver were not analyzed. Therefore, analysis of the mechanisms involved in the interaction among organs in hepatic steatosis must be performed in future studies.

The liver plays an important role as an organ that stores and supplies energy. Liver glycogen is used to replenish glucose circulating in the blood continuously [38] and produce ATP in muscles, especially during exercise [39]. Furthermore, glycogen can contribute to approximately 5% of the healthy liver weight [38], and sustained exercise is impossible without liver glycogen activity [40]. In this study, resistance exercise reduced hepatic steatosis, but liver weight tended to be high. Although glycogen storage in the liver was not analyzed, we suggested that changes to a healthy liver through hepatic steatosis reduction and liver weight maintenance might indicate improvement in the glycogen storage capacity of the liver. Therefore, confirming whether resistance exercise leads to an increase in the glycogen storage capacity of the liver by analyzing liver glycogen levels in future studies is warranted.

In summary, both aerobic and resistance exercises were found to be effective in improving fat mass in adipose tissue and hepatic steatosis, even though reduction in body weight and liver weight was not achieved since dietary conversion to a low-fat or low-calorie diet was not performed. Moderate-intensity aerobic exercise without dietary restriction improved liver TG levels and fat accumulation-related factors, while reducing fat mass. Similar to aerobic exercise, moderate-intensity resistance exercise effectively improved fat accumulation-related factors and relieved hepatic steatosis, while reducing fat mass. Therefore, resistance exercise, such as aerobic exercise (which is generally recommended), can be recommended for obese or musculoskeletal disorder patients who have difficulty in performing aerobic exercise to alleviate NAFLD.

## 5. Conclusions

The results of this study showed that both aerobic and resistance exercise were effective in improving fat mass and hepatic lipolysis of HFD-induced obese mice. Therefore, the results of this study suggested that a sustainable type of exercise selected based on the fitness level, disease type, musculoskeletal disorder status, and preference of the patients is the best exercise intervention for alleviating NAFLD.
